# Use of antibiotics in children younger than two years in eight countries: a prospective cohort study

**DOI:** 10.2471/BLT.16.176123

**Published:** 2016-11-03

**Authors:** Elizabeth T Rogawski, James A Platts-Mills, Jessica C Seidman, Sushil John, Mustafa Mahfuz, Manjeswori Ulak, Sanjaya K Shrestha, Sajid Bashir Soofi, Pablo Penataro Yori, Estomih Mduma, Erling Svensen, Tahmeed Ahmed, Aldo AM Lima, Zulfiqar A Bhutta, Margaret N Kosek, Dennis R Lang, Michael Gottlieb, Anita KM Zaidi, Gagandeep Kang, Pascal O Bessong, Eric R Houpt, Richard L Guerrant

**Affiliations:** a Division of Infectious Diseases and International Health, University of Virginia, PO Box 801379, Carter Harrison Research Bldg MR-6, 345 Crispell Drive, Room 2520, Charlottesville, Virginia 22908-1379, United States of America (USA).; b Fogarty International Center, National Institutes of Health, Bethesda, USA.; c Christian Medical College, Vellore, India.; d International Centre for Diarrhoeal Disease Research, Dhaka, Bangladesh.; e Institute of Medicine, Tribhuvan University, Kathmandu, Nepal.; f Walter Reed/AFRIMS Research Unit, Kathmandu, Nepal.; g Aga Khan University, Karachi, Pakistan.; h Bloomberg School of Public Health, Johns Hopkins University, Baltimore, USA.; i Haydom Lutheran Hospital, Haydom, United Republic of Tanzania.; j Haukeland University Hospital, Bergen, Norway.; k Clinical Research Unit and Institute of Biomedicine, Federal University of Ceara, Fortaleza, Brazil.; l Foundation for the National Institutes of Health, Bethesda, USA.; m University of Venda, Thohoyandou, South Africa.; Correspondence to Elizabeth T Rogawski (email: etr5m@virginia.edu).

## Abstract

**Objective:**

To describe the frequency and factors associated with antibiotic use in early childhood, and estimate the proportion of diarrhoea and respiratory illnesses episodes treated with antibiotics.

**Methods:**

Between 2009 and 2014, we followed 2134 children from eight sites in Bangladesh, Brazil, India, Nepal, Pakistan, Peru, South Africa and the United Republic of Tanzania, enrolled in the MAL-ED birth cohort study. We documented all antibiotic use from mothers’ reports at twice-weekly visits over the children’s first two years of life. We estimated the incidence of antibiotic use and the associations of antibiotic use with child and household characteristics. We described treatment patterns for diarrhoea and respiratory illnesses, and identified factors associated with treatment and antibiotic class.

**Findings:**

Over 1 346 388 total days of observation, 16 913 courses of antibiotics were recorded (an incidence of 4.9 courses per child per year), with the highest use in South Asia. Antibiotic treatment was given for 375/499 (75.2%) episodes of bloody diarrhoea and for 4274/9661 (44.2%) episodes of diarrhoea without bloody stools. Antibiotics were used in 2384/3943 (60.5%) episodes of fieldworker-confirmed acute lower respiratory tract illness as well as in 6608/16742 (39.5%) episodes of upper respiratory illness. Penicillins were used most frequently for respiratory illness, while antibiotic classes for diarrhoea treatment varied within and between sites.

**Conclusion:**

Repeated antibiotic exposure was common early in life, and treatment of non-bloody diarrhoea and non-specific respiratory illnesses was not consistent with international recommendations. Rational antibiotic use programmes may have the most impact in South Asia, where antibiotic use was highest.

## Résumé

**Objectif:**

Décrire la fréquence et les facteurs associés à l'utilisation d'antibiotiques chez les jeunes enfants et estimer la proportion d'épisodes de diarrhée et de maladies respiratoires traités avec des antibiotiques.

**Méthodes:**

De 2009 à 2014, nous avons suivi 2134 enfants qui participaient à l'étude de cohorte de naissance MAL-ED sur huit sites en Afrique du Sud, au Bangladesh, au Brésil, en Inde, au Népal, au Pakistan, au Pérou et en République-Unie de Tanzanie. Nous avons noté l'utilisation de tous les antibiotiques déclarés par les mères lors de consultations bi-hebdomadaires pendant les deux premières années de vie des enfants. Nous avons estimé l'incidence du recours aux antibiotiques ainsi que les associations entre utilisation d'antibiotiques et caractéristiques des enfants et des foyers. Nous avons décrit les habitudes de traitement de la diarrhée et des maladies respiratoires et avons identifié les facteurs associés aux traitements et aux classes d'antibiotiques.

**Résultats:**

Sur 1 346 388 jours d'observation au total, 16 913 traitements aux antibiotiques ont été enregistrés (incidence de 4,9 traitements par enfant et par an), la plus forte utilisation ayant été observée en Asie du Sud. Un traitement antibiotique a été administré pour 375/499 (75,2%) épisodes de diarrhée sanglante et pour 4274/9661 (44,2%) épisodes de diarrhée sans présence de sang dans les selles. Des antibiotiques ont été utilisés pour 2384/3943 (60,5%) épisodes de maladie aiguë des voies respiratoires inférieures confirmée par un professionnel sur le terrain ainsi que pour 6608/16 742 (39,5%) épisodes de maladie des voies respiratoires supérieures. Les pénicillines étaient les plus fréquemment utilisées pour combattre les maladies respiratoires, tandis que les classes d'antibiotiques utilisées pour traiter la diarrhée variaient selon les sites et au sein d'un même site.

**Conclusion:**

L'exposition répétée aux antibiotiques à un jeune âge était courante et le traitement de la diarrhée sans présence de sang dans les selles et de maladies respiratoires non spécifiques ne respectait pas les recommandations internationales. C'est en Asie du Sud, où l'usage des antibiotiques était le plus important, que les programmes d'utilisation rationnelle des antibiotiques pourraient avoir le plus fort impact.****

## Resumen

**Objetivo:**

Describir la frecuencia y los factores relacionados con el uso de antibióticos en la primera infancia y estimar la proporción de los episodios de diarrea y enfermedades respiratorias tratados con antibióticos.

**Métodos:**

Entre 2009 y 2014, se realizó el seguimiento de 2 134 niños de ocho lugares en Bangladesh, Brasil, India, Nepal, Pakistán, Perú, la República Unida de Tanzania y Sudáfrica inscritos en el estudio de cohortes en el nacimiento MAL-ED. Se documentó el uso de todos los antibióticos de los informes de las madres en las visitas dos veces por semana a lo largo de los dos primeros años de vida de los niños. Se estimó la incidencia del uso de antibióticos y las asociaciones del uso de antibióticos con características familiares y con niños. Se describieron los patrones de tratamiento para la diarrea y las enfermedades respiratorias, y se identificaron los factores relacionados con las clases de tratamientos y de antibióticos.

**Resultados:**

De un total de 1 346 388 días de observación, se registraron 16 913 tratamientos con antibióticos (una incidencia de 4,9 tratamientos por niño al año), siendo el mayor uso en el sur de Asia. Se empleó tratamiento con antibióticos en 375/499 (75,2%) episodios de diarrea hemorrágica y en 4 274/9 661 (44,2%) episodios de diarrea sin deposiciones hemorrágicas. Se usaron antibióticos en 2 384/3 943 (60,5%) episodios de enfermedad respiratoria aguda de las vías bajas confirmadas por los investigadores, así como en 6 608/16 742 (39,5%) episodios de enfermedad respiratoria de las vías altas. Las penicilinas se usaron más frecuentemente para las enfermedades respiratorias, mientras que los antibióticos para el tratamiento de la diarrea variaron entre los distintos lugares.

**Conclusión:**

La exposición repetida a antibióticos fue común en los primeros años de vida, y el tratamiento de diarrea no hemorrágica y de enfermedades respiratorias no específicas no fue coherente con las recomendaciones internacionales. Los programas de uso racional de antibióticos pueden tener el mayor efecto en el sur de Asia, donde el uso de antibióticos fue el más alto.

## ملخص

**الغرض:**

وصف وتيرة إعطاء المضادات الحيوية للأطفال في مراحلهم العمرية المبكرة والعوامل المرتبطة بذلك، وتقدير نسبة حالات الإصابة بالإسهال وأمراض الجهاز التنفسي التي عولجت بالمضادات الحيوية.

**الطريقة:**

قمنا في الفترة ما بين عامي 2009 و 2014 بمتابعة 2134 طفلاً من ثماني مواقع في بنغلاديش والبرازيل والهند ونيبال وباكستان وبيرو وجنوب أفريقيا وجمهورية تنزانيا المتحدة، الذين تم تسجيلهم في دراسة الأترابية الخاصة بالمواليد MAL-ED. وقمنا بتوثيق جميع حالات تناول المضادات الحيوية من بلاغات الأمهات في الزيارات الأسبوعية التي تحدث مرتين طوال العامين الأولين للطفل. كما قمنا بتقدير عدد حالات تناول المضادات الحيوية والروابط بين تناول المضادات الحيوية مع خصائص الطفل والأسرة. وبذلك قدمنا وصفًا لأنماط العلاج للحالات المصابة بالإسهال وأمراض الجهاز التنفسي، وحددنا العوامل المرتبطة بفئة العلاج والمضاد الحيوي.

**النتائج:**

على مدار 1346388 من إجمالي أيام الرصد والملاحظة، تم تسجيل إعداد 16913 خطة علاجية بالمضادات الحيوية (بمعدل إعداد 4.9 خطة علاجية لكل طفل في العام الواحد)، مع ملاحظة أن معدل تناول المضادات الحيوية هو الأعلى في جنوب آسيا. تم إعطاء العلاج بالمضادات الحيوية بمعدل 375/499 (75.2‏%) للحالات المصابة بالإسهال الدموي وبمعدل 4274/9661 (44.2‏%) للحالات المصابة بالإسهال من دون نزول براز دموي. وتم تناول المضادات الحيوية في 2348/3943 (60.5‏%) من حالات الإصابة المؤكدة بعدوى القناة التنفسية السفلية الحاد وكذلك في 6608/16742 (39.5‏%) من حالات الإصابة المؤكدة بعدوى القناة التنفسية العلوية لدى العاملين الميدانيين. تم إعطاء أدوية البنسلين في معظم الأحيان لعلاج أمراض الجهاز التنفسي، بينما تباينت فئات المضادات الحيوية لعلاج الإسهال داخل وبين المواقع المختلفة.

**الاستنتاج:**

لقد كان التعرض المتكرر للمضادات الحيوية شائعًا في مرحلة عمرية مبكرة، ولم يكن علاج الإسهال غير الدموي وأمراض الجهاز التنفسي غير المحددة متناسقًا مع التوصيات الدولية. ولعل برامج تناول المضادات الحيوية المنطقية هي ما حقق أكبر الأكبر في جنوب آسيا، حيث كان معدل تناول المضادات الحيوية هو الأعلى.

## 摘要

**目的:**

旨在描述幼儿期抗生素使用频率及相关因素，并评估使用抗生素进行治疗的腹泻和呼吸道疾病病例的比例。

**方法:**

在 2009 年至 2014 年期间，我们对巴基斯坦、巴西、秘鲁、孟加拉国、南非、尼泊尔、坦桑尼亚联合共和国以及印度八个地区报名参加营养不良和肠道疾病联盟 (MAL-ED) 出生群组研究的 2134 名儿童进行了跟踪调查。 我们在儿童出生后两年内对其进行每周两次的访问，通过其母亲的叙述记录了全部抗生素使用情况。 我们对抗生素使用率以及抗生素使用与儿童和家庭特征之间的关联进行了评估。 我们描述了腹泻和呼吸道疾病的治疗模式，并确定了与治疗和抗生素种类有关的因素。

**结果:**

经过共 1 346 388 天的观察，我们记录了 16 913 个抗生素使用案例（每名儿童每年平均使用 4.9 次），其中抗生素在南亚的使用率最高。 出血性腹泻的抗生素治疗比例为 375/499 (75.2%)，无血便性腹泻的抗生素治疗比例为 4274/9661 (44.2%)。 在经实地调查员确认的急性下呼吸道疾病案例中，抗生素使用比例为 2384/3943 (60.5%)，上呼吸道疾病案例中，抗生素使用比例为 6608/16742 (39.5%)。盘尼西林最常用于呼吸道疾病治疗，腹泻治疗用抗生素的种类在同一地区内以及不同地区间均有所不同。

**结论:**

反复接触抗生素在童年时期很常见，并且非出血性腹泻和非特异性呼吸道疾病的疗法与国际建议不一致。 合理使用抗生素项目或许在南非会产生最大影响，在该地区，抗生素的使用率最高。****

## Резюме

**Цель:**

Описать частоту употребления антибиотиков в раннем детстве и факторы, связанные с этим, и определить долю случаев диареи и заболеваний дыхательных путей, для лечения которых использовались антибиотики.

**Методы:**

В период между 2009 и 2014 годами авторы осуществляли наблюдение за 2134 детьми из восьми локаций в Бангладеш, Бразилии, Индии, Непале, Объединенной Республике Танзания, Пакистане, Перу и Южной Африке, участвующих в исследовании возрастной группы MAL-ED. Все случаи приема антибиотиков были задокументированы со слов матерей работниками местного центра, совершавшими визит два раза в неделю в течение первых двух лет жизни ребенка. Авторы определили частотность приема антибиотиков и связи между приемом антибиотиков детьми и особенностями домашнего хозяйства. Были описаны модели лечения диареи и заболеваний дыхательных путей, а также определены факторы, связанные с лечением и классом антибиотиков.

**Результаты:**

За 1 346 388 суммарных дней наблюдения было зарегистрировано 16 913 курсов лечения антибиотиками (частотность составила 4,9 курса на ребенка в год), большая доля которых пришлась на Южную Азию. Лечение антибиотиками было прописано в 375 из 499 случаев диареи с кровью (75,2%) и в 4274 из 9661 случая диареи без кровавых примесей (44,2%). Антибиотики применялись в 2384 из 3943 случаев острого заболевания нижних дыхательных путей (60,5%), подтвержденного работниками местного центра, а также в 6608 из 16 742 случаев заболевания верхних дыхательных путей (39,5%). Чаще других для лечения заболеваний дыхательных путей использовались пенициллины, в то время как класс антибиотиков для лечения диареи различался как в пределах одной локации, так и между локациями.

**Вывод:**

В раннем возрасте было распространено неоднократное воздействие антибиотиков, а лечение диареи без следов крови и неспецифических заболеваний дыхательных путей проводилось не в соответствии с международными рекомендациями. Программы рационального использования антибиотиков, возможно, оказывают наибольшее влияние в Южной Азии, где процент применения антибиотиков был наиболее высок.****

## Introduction

Antibiotics can be a lifesaving treatment for children with bacterial infections and are the most commonly prescribed therapy among all medications given to children.^1^ However, antibiotics can also result in adverse events, drug toxicity and detrimental effects on the gut microbiota^2^^,^^3^ and enteric immune system.^4^^,^^5^ Furthermore, both at the individual and population levels, antibiotic overuse drives the development and transmission of antimicrobial resistance.^1^^,^^6^ International guidelines for the treatment of childhood illnesses recommend antibiotic treatment for diarrhoea with bloody stools and for acute lower respiratory tract infections, but not for non-bloody diarrhoea and for upper respiratory infections.^7^^,^^8^ Interventions to promote rational antibiotic use are critical for preserving the effectiveness of available drugs.^9^^–^^11^ Conversely, in low-resource settings, the high burden of bacterial causes of diarrhoea in children^12^^,^^13^ has led to proposals for antibiotics to be used more widely for the treatment of diarrhoea even in the absence of dysentery.^14^^–^^16^ Antibiotics may also be a potential intervention for malnutrition and environmental enteropathy.^17^

Differences in antibiotic use practices around the world reflect differences in local medication policies, in barriers to access to care and in the preferences of health-care providers and mothers. The availability of antibiotics without a doctor’s prescription varies,^6^^,^^18^ and laws to limit access to antibiotics are often poorly enforced.^6^^,^^19^^–^^22^ In some settings, drug shortages may be a major limiter of antibiotic use.^18^^,^^23^ Cultural preferences, such as high demand by mothers, also influence patterns of antibiotic use.^19^^,^^22^^,^^24^^,^^25^ Even when health-care providers are aware of the appropriate indications for antibiotics, there can be differences between knowledge and practice.^26^^,^^27^

Many studies of antibiotic use have been conducted in various health-care settings^22^^,^^28^^–^^30^ and in cross-sectional community-based surveys.^31^^–^^35^ Nevertheless, high-resolution, systematic assessments of antibiotic use in prospective, observational cohort studies have not been reported. MAL-ED (Etiology, Risk Factors, and Interactions of Enteric Infections and Malnutrition and the Consequences for Child Health and Development Project) was a multisite birth cohort study conducted in eight sites in different countries of South America, sub-Saharan Africa and Asia.^36^ This study included community-based surveillance for antibiotic use and provides an opportunity to compare antibiotic use patterns across diverse low-resource sites. We aimed to describe the frequency of antibiotic use by children in the first two years of life; determine the characteristics associated with antibiotic use; and estimate the proportions of diarrhoea and respiratory illness episodes treated with antibiotics, as reported by mothers in the MAL-ED study.

## Methods

The MAL-ED study design^36^ and cohort characteristics have been previously described.^37^ Briefly, the study was conducted at sites in eight different countries: Dhaka (Bangladesh), Fortaleza (Brazil), Vellore (India), Bhaktapur (Nepal), Naushahro Feroze (Pakistan), Loreto (Peru), Venda (South Africa) and Haydom (United Republic of Tanzania). Healthy children were enrolled between November 2009 and February 2012 within 17 days of birth. Two-year follow-up for all enrolled children was completed in February 2014. The criteria for enrolment were children without severe or chronic conditions, enteropathy or hospitalization, and enrolment weight ≥ 1500 g.

Surveillance for illnesses and antibiotic use was conducted twice per week by fieldworkers at home visits until the child was two years of age or was lost to follow-up. Children were referred to locally available care, generally a local clinic, when ill.^38^ Fieldworkers asked the mother (or other caregiver) to report all oral or injected antibiotics given to their child on each day since the previous visit and to show the medication packaging to confirm the antibiotic and class. If packaging were not available, fieldworkers documented antibiotic use from any paperwork provided by health-care providers. When a mother reported seeking medical care or medications for their child, fieldworkers recorded separately prescribed medicines in medical care report forms. Socioeconomic characteristics were assessed through twice-yearly questionnaires. 

To validate mothers’ reports of antibiotic use, we randomly selected 4409 of the fieldworkers’ medical care report forms (including at least 200 records of antibiotics per site) and extracted all antibiotic information. We assessed the concordance between mother-reported antibiotic use and antibiotic use as documented on the medical care report forms.

All sites received ethical approval from their respective government, local institution and collaborating institution ethical review boards. We obtained informed consent from the mother of each child.

### Data and definitions

We counted distinct antibiotic courses when separated by at least two antibiotic-free days. The results were insensitive to an alternative definition using three antibiotic-free days; only 580 (3.4%) of 16 913 courses occurred within three days. The duration of antibiotic courses was defined as the total number of days on which antibiotics were received, assuming antibiotics were not received on missed surveillance days (2.0% of all surveillance days). A child was classified as exposed to high antibiotic use if he or she received more than or equal to the median number of courses received by children at his or her study site in the first two years of life.

We based illness definitions on Integrated Management of Childhood Illness guidelines.^7^ Non-bloody diarrhoea was defined as mother’s reports of three or more loose stools in 24 hours. Bloody diarrhoea was defined as mother’s report of at least one loose stool with visible blood.^38^ Respiratory illness was defined as cough or shortness of breath. Acute lower respiratory tract illness was defined as cough or shortness of breath with a rapid respiratory rate determined by fieldworkers (defined by the average of two measurements per day that were: > 60 breaths per minute when the child was < 2 months old; > 50 breaths per minute at age 2 months to 1 year; and > 40 breaths per minute at age ≥ 1 year).^38^ If antibiotics were taken during any day of the illness episode, the episode was classified as treated with antibiotics.

Socioeconomic status was described using the child’s average score on the WAMI index based on: household access to improved water and sanitation; wealth measured by eight household assets; mother’s education; and monthly household income.^39^ Crowding was defined as the mean number of people per room. Improved water and sanitation were defined following World Health Organization (WHO) guidelines.^40^

### Analysis

We calculated the incidence of antibiotic use as the number of courses divided by the number of at-risk surveillance days. The incidence over the first two years of life was estimated using a pooled logistic regression model with a restricted quadratic spline^41^ for age with seven knots. Cumulative incidence curves were constructed non-parametrically as the inverse of Kaplan–Meier estimates.

We adjusted for the following: study site; the proportion of days ill with diarrhoea, cough, fever, vomiting and fieldworker-confirmed acute lower respiratory infection in the first two years of life; and the interaction between this proportion and study site. To estimate the associations between overall antibiotic use and child and household characteristics we used linear regression for the proportion of days on antibiotics and log-binomial regression for risk of high antibiotic use.

We then described the frequency of treatment for diarrhoea and respiratory illnesses. We estimated the associations between the characteristics of those episodes and antibiotic treatment using log-binomial regression, adjusting for study site and other episode characteristics. We also accounted for correlations between episodes in the same child using generalized estimating equations with a robust variance estimator. Among treated episodes, we used these log-binomial models to estimate the associations between antibiotic class chosen and episode and child characteristics.

## Results

### Antibiotic use

We included 2134 children in the MAL-ED cohort who were surveyed for antibiotic use for any illness on at least one day in the first two years of life. Over a mean of 631 days of observation per child (1 346 388 total days of observation), 16 913 courses (100 342 total days) of antibiotics were recorded. This corresponded to an overall average antibiotic use of 4.9 courses per child per year. The median duration of antibiotic courses was 5 days (interquartile range: 3 to 7). Extended courses of antibiotics were rare; only 53 (0.3%) courses had durations longer than 1 month.

**Fig. 1:**
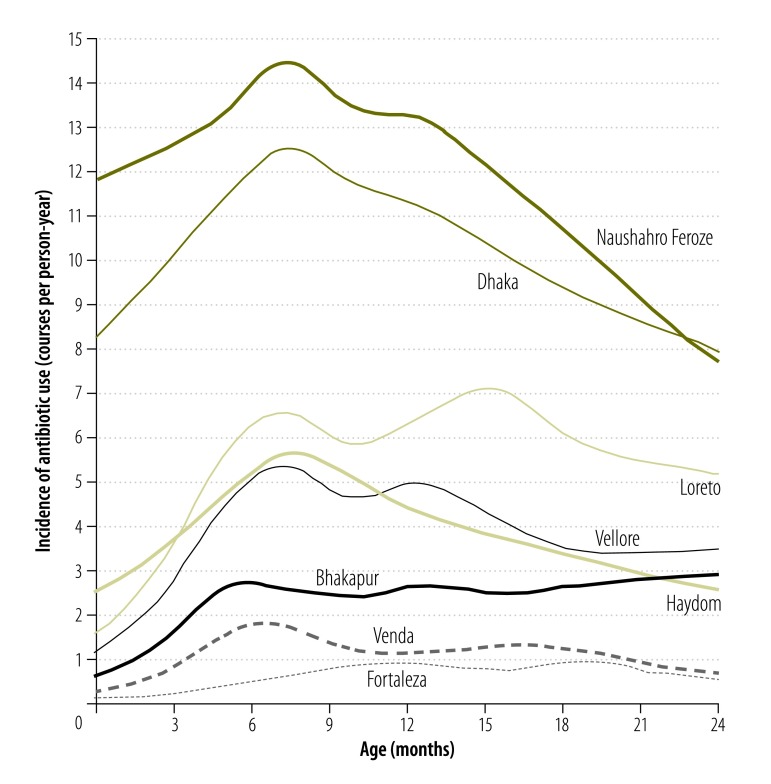
Incidence of antibiotic use in the first two years of life by study site among 2134 children in the MAL-ED birth cohort, 2009–2014

The magnitude of use differed across the eight sites (Fig. 1). Frequency of use was highest at the site in Naushahro Feroze (an average of 11.9 courses per child-year) and in Dhaka (10.3 courses per child-year). In contrast, the use was ≤ 1.0 course per child-year in Fortaleza and Venda, respectively. Antibiotic use peaked between 6 and 12 months of age in all sites, and peaked again in the second year of life in Loreto, Vellore and Fortaleza.

MAL-ED: Etiology, Risk Factors, and Interactions of Enteric Infections and Malnutrition and the Consequences for Child Health and Development Project.

Notes: Incidence was calculated using restricted quadratic splines for age.^41^ The total number of antibiotic courses was 16913. Number of antibiotic courses at study sites: Dhaka (Bangladesh) *n* = 4062; Fortaleza (Brazil) *n* = 235; Vellore (India) *n* = 1730; Bhaktapur (Nepal) *n* = 1065; Naushahro Feroze (Pakistan) *n* = 5142; Loreto (Peru) *n* = 2447; Venda (South Africa) *n* = 534; Haydom (United Republic of Tanzania) *n* = 1698.

**Fig. 2:**
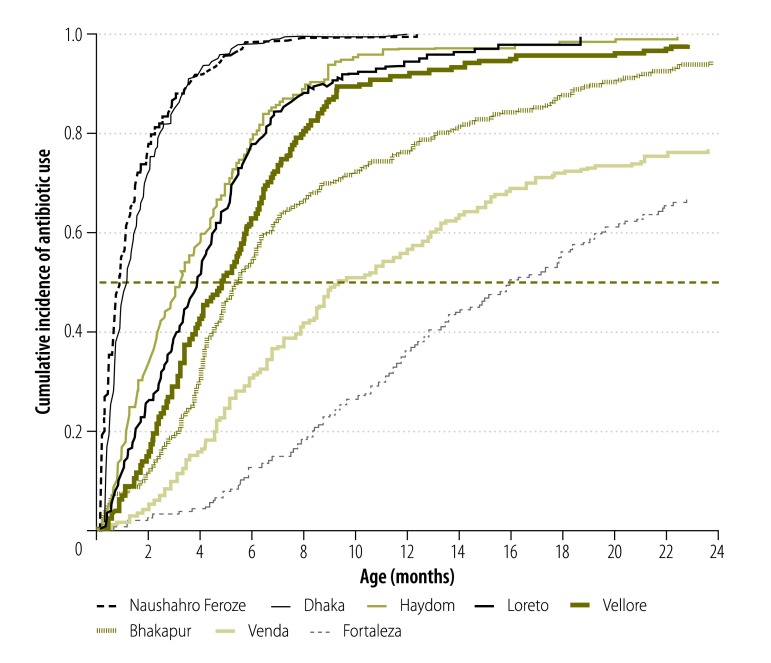
Cumulative incidence of first antibiotic use in the first two years of life by study site among 2134 children in the MAL-ED birth cohort, 2009–2014

Early antibiotic use was common even in the first 6 months of life. In Dhaka and Naushahro Feroze, more than 98.0% of children followed until at least age 6 months had received antibiotics by that age (Fig. 2). More than half of children had received antibiotics by age 6 months in Bhaktapur, Haydom, Loreto and Vellore. Children in Naushahro Feroze were exposed to antibiotics on 32 345 (17.5%) of 152 176 observed child-days in the first two years of life, which corresponds to more than 4 months of cumulative antibiotic treatment. This proportion was even higher in the first 6 months of life (9383 child-days; 19.5%). The days of treatment in the first two years of life was lower in other sites, ranging from 1823 (1.3%) of 138 060 observed child-days in Fortaleza to 25 663 (15.5%) of 140 237 child-days in Dhaka.

MAL-ED: Etiology, Risk Factors, and Interactions of Enteric Infections and Malnutrition and the Consequences for Child Health and Development Project.

Notes: The black dotted line indicates age at each study site where 50% of children received at least one course of antibiotics. Incidence was calculated using restricted quadratic splines for age.^41^ The total number of antibiotic courses was 16913. Number of antibiotic courses at study sites: Dhaka (Bangladesh) *n* = 4062; Fortaleza (Brazil) *n* = 235; Vellore (India) *n* = 1730; Bhaktapur (Nepal) *n* = 1065; Naushahro Feroze (Pakistan) *n* = 5142; Loreto (Peru) *n* = 2447; Venda (South Africa) *n* = 534; Haydom (United Republic of Tanzania) *n* = 1698.

A total of 1741 children (81.6%) remained under surveillance until at least two years of age. More boys (543/888; 61.2%) than girls (436/853; 51.1%) received at or above the site-specific median number of antibiotic courses (Table 1). Adjusting for the proportion of days ill, the risk of high antibiotic use was 13% greater among boys compared with girls (risk ratio, RR: 1.13; 95% confidence interval, CI: 0.99 to 1.28). The association between antibiotic use and sex was driven by Naushahro Feroze (RR: 1.44; 95% CI: 1.02 to 2.03) and Bhaktapur (RR: 1.37; 95% CI: 0.9 to 1.97), with no associations at the other country sites. Socioeconomic status and income were associated with small increases in overall antibiotic use (Table 1).

**Table 1:** Associations between antibiotic use in the first two years of life and characteristics of children and their families among 1741 children who remained in the MAL-ED cohort for at least two years, 2009–2014

Characteristic	All children	Children with high antibiotic use^a^	Difference in proportion of days on antibiotics^b^ (95% CI)	RR for high antibiotic use^a,b^ (95% CI)
**Child’s sex, no. of children**				
Female	853	436 (51.1)	0.00 (ref)	1.00 (ref)
Male	888	543 (61.2)	0.01 (0.00 to 0.01)	1.13 (0.99 to 1.28)
**Socioeconomic status (per 0.5 increase in WAMI score),^c^ mean (SD)**	0.57 (0.22)	0.57 (0.22)	0.02 (0.01 to 0.03)	1.10 (0.86 to 1.40)
**Household monthly income,^c^ no. of children**				
Below site-specific median	1093	615 (56.3)	0.00 (ref)	1.00 (ref)
At or above site-specific median	647	364 (56.3)	0.01 (0.00 to 0.01)	1.02 (0.90 to 1.17)
**Mother’s age (per 5 year increase),^c^ mean (SD)**	26 (5.9)	26 (6.0)	0.00 (0.00 to 0.00)	0.99 (0.94 to 1.05)
**Mother’s education,^c^ no. of children**				
< 6 years	637	347 (54.5)	0.00 (ref)	1.00 (ref)
≥ 6 years	1102	630 (57.2)	0.00 (0.00 to 0.01)	1.02 (0.88 to 1.19)
**Crowding (per 1 person increase in mean people per household room),^c^ mean (SD)**	2.4 (1.5)	2.4 (1.5)	0.00 (0.00 to 0.00)	0.98 (0.93 to 1.04)
**Birth weight,^c^ no. of children**				
Normal	1275	711 (55.8)	0.00 (ref)	1.00 (ref)
Low	295	166 (56.3)	0.00 (0.00 to 0.01)	1.05 (0.86 to 1.29)
**Age at first milk or solids introduced (per 1 month increase),^c^ mean (SD)**	2.7 (2.0)	2.7 (2.0)	0.00 (0.00 to 0.00)	1.00 (0.97 to 1.04)
**Age at stopping all breastfeeding (per 1 month increase),^c^ mean (SD)**	21.0 (7.6)	20.9 (7.7)	0.00 (0.00 to 0.00)	1.00 (0.99 to 1.01)
**Sanitation,^c^ no. of children**				
Unimproved	496	281 (56.7)	0.00 (ref)	1.00 (ref)
Improved	1243	697 (56.1)	0.01 (0.00 to 0.02)	1.09 (0.86 to 1.38)
**Water source,^c^ no. of children**				
Unimproved	171	100 (58.5)	0.00 (ref)	1.00 (ref)
Improved	1568	878 (56.0)	0.00 (−0.01 to 0.01)	0.94 (0.68 to 1.28)

CI: confidence interval; MAL-ED: Etiology, Risk Factors, and Interactions of Enteric Infections and Malnutrition and the Consequences for Child Health and Development Project; ref: reference; RR: risk ratio; SD: standard deviation; WAMI: water and sanitation, assets, maternal education and income.

^a^ High antibiotic use was defined as receiving at or above the site-specific median number of antibiotic courses in the first two years of life.

^b^ In the first two years of life among children followed for at least two years. All estimates were adjusted for site; proportion of days ill with diarrhoea, cough, fever, vomiting or fieldworker-confirmed acute lower respiratory tract illness in the first 2 years of life; and the interaction between the proportion of days ill and study site.

^c^ Data on income, mother’s age and education, crowding, sanitation and water source missing for 2 children; income missing for 1 child; birth weight missing for 171 children; age at first milk or solids missing for 17 children; age at stopping all breastfeeding missing for 8 children.

### Diarrhoea treatment

A total of 10 161 diarrhoea episodes were recorded among 1201 of the children; 4649 (45.8%) episodes were treated with antibiotics (Table 2). The use of antibiotics for the treatment of diarrhoea varied across sites from 10.6% of 180 episodes in Fortaleza to 59.1% of 3212 episodes in Naushahro Feroze.

**Table 2:** Proportion of illness episodes treated with antibiotics among children in the MAL-ED cohort, by study site, 2009–2014

Study site	Total no. of children	Episodes of diarrhoea^a^						Episodes of respiratory illness^a^				
		Non-bloody			Bloody			Non-specific respiratory tract illness			Acute lower respiratory tract illness	
		Total no.	Antibiotic treated, no. (%)		Total no.	Antibiotic treated, no. (%)		Total no.	Antibiotic treated, no. (%)		Total no.	Antibiotic treated, no. (%)
Bhaktapur (Nepal)	240	1 027	284 (27.7)		50	42 (84.0)		1 873	379 (20.2)		442	221 (50.0)
Dhaka (Bangladesh)	265	1 597	914 (57.2)		73	58 (79.5)		4 284	2 391 (55.8)		214	184 (86.0)
Fortaleza (Brazil)	233	176	17 (9.7)		4	2 (50.0)		393	135 (34.4)		38	18 (47.4)
Haydom (United Republic of Tanzania)	262	539	260 (48.2)		84	62 (73.8)		1 285	799 (62.2)		114	79 (69.3)
Loreto (Peru)	303	3 111	1 817 (58.4)		101	82 (81.2)		1 720	777 (45.2)		2 072	1 386 (66.9)
Naushahro Feroze (Pakistan)	277	1 988	695 (35.0)		114	97 (85.1)		3 895	1 332 (34.2)		237	158 (66.7)
Vellore (India)	251	913	220 (24.1)		61	28 (45.9)		2 325	539 (23.2)		698	278 (39.8)
Venda (South Africa)	303	311	67 (21.5)		12	4 (33.3)		967	256 (26.5)		128	60 (46.9)
**All sites**	**2 134**	**9 662**	**4 274 (44.2)**		**499**	**375 (75.2)**		**16 742**	**6 608 (39.5)**		**3 943**	**2 384 (60.5)**

MAL-ED: Etiology, Risk Factors, and Interactions of Enteric Infections and Malnutrition and the Consequences for Child Health and Development Project.

^a^ 1201 children had one or more episodes of diarrhoea; 1672 children had one or more episodes of respiratory illness.

Mothers reported bloody stools in 499 (4.9%) diarrhoea episodes. A higher proportion of episodes of bloody diarrhoea (375; 75.2%) were treated with antibiotics than those without bloody stools (4274/9661; 44.2%; Table 3). Adjusting for study site and other characteristics of illness episodes, the risk ratio of antibiotic treatment was 1.50 (95% CI: 1.40 to 1.64) for episodes with bloody stools. Greater age at episode, duration, number of loose stools, and presence of fever, dehydration and vomiting were all independently associated with an increased risk of antibiotic treatment.

**Table 3:** Characteristics of illness episodes and their association with antibiotic treatment among children in the MAL-ED cohort, 2009–2014

Characteristics	Episodes of diarrhoea^a^					Episodes of respiratory illnesses^a^			
	Total no.	Antibiotic treated, no. (%)	Crude risk ratio^b^ (95% CI)	Adjusted risk ratio^b,c^ (95% CI)		Total no.	Antibiotic treated, no. (%)	Crude risk ratio^b^ (95% CI)	Adjusted risk ratio^b,c^ (95% CI)
**Demographic characteristics**									
Child’s sex									
Male	5 264	2 471 (46.9)	1.00 (ref)	1.00 (ref)		10 666	4 793 (44.9)	1.00 (ref)	1.00 (ref)
Female	4 897	2 178 (44.5)	0.94 (0.90 to 0.99)	0.95 (0.90 to 1.00)		10 019	4 199 (41.9)	0.93 (0.89 to 0.96)	0.94 (0.91 to 0.98)
**Socioeconomic status, per 0.5 WAMI increase **	10 161	NA^d^	1.18 (1.09 to 1.28)	1.19 (1.10 to 1.29)		20 685	NA^d^	1.07 (0.99 to 1.15)	1.10 (1.03 to 1.17)
**Illness characteristics**									
**Age at illness episode, months**									
< 6	2 746	1 092 (39.8)	1.00 (ref)	1.00 (ref)		5 770	2 458 (42.6)	1.00 (ref)	1.00 (ref)
6–12	3 148	1 490 (47.3)	1.23 (1.16 to 1.30)	1.25 (1.18 to 1.32)		5 929	2 684 (45.3)	1.08 (1.04 to 1.12)	1.05 (1.00 to 1.09)
12–24	4 267	2 067 (48.4)	1.26 (1.19 to 1.34)	1.41 (1.33 to 1.49)		8 986	3 850 (42.8)	1.01 (0.97 to 1.05)	1.02 (0.98 to 1.06)
**Duration, days**									
1–6	8 429	3 531 (41.9)	1.00 (ref)	1.00 (ref)		11 456	3 493 (30.5)	1.00 (ref)	1.00 (ref)
7–13	1 380	846 (61.3)	1.46 (1.40 to 1.53)	1.22 (1.16 to 1.28)		5 936	3 126 (52.7)	1.80 (1.73 to 1.88)	1.54 (1.48 to 1.60)
≥ 14	352	272 (77.3)	1.64 (1.55 to 1.74)	1.26 (1.18 to 1.35)		3 296	2 373 (72.1)	2.38 (2.28 to 2.48)	1.77 (1.69 to 1.84)
**Fever^e^**									
None	6 889	2 483 (36.0)	1.00 (ref)	1.00 (ref)		10 715	2 982 (27.8)	1.00 (ref)	1.00 (ref)
Mother-reported	2 853	1 872 (65.6)	1.66 (1.58 to 1.74)	1.48 (1.42 to 1.55)		8 223	4 805 (58.4)	2.02 (1.94 to 2.10)	1.76 (1.70 to 1.83)
Confirmed	416	294 (70.7)	1.95 (1.83 to 2.08)	1.63 (1.51 to 1.75)		1 747	1 205 (69.0)	2.44 (2.33 to 2.55)	2.06 (1.96 to 2.16)
**Diarrhoea-specific characteristics^e^**									
Bloody stools									
No	9 661	4 274 (44.2)	1.00 (ref)	1.00 (ref)		NA	NA	NA	NA
Yes	499	375 (75.2)	1.57 (1.47 to 1.67)	1.50 (1.40 to 1.64)		NA	NA	NA	NA
**Dehydration**									
No	9 165	3 909 (42.7)	1.00 (ref)	1.00 (ref)		NA	NA	NA	NA
Yes	996	740 (74.3)	1.54 (1.46 to 1.62)	1.12 (1.07 to 1.18)		NA	NA	NA	NA
**Vomiting, days**									
0	7 409	3 023 (40.8)	1.00 (ref)	1.00 (ref)		NA	NA	NA	NA
1	1 197	635 (53.1)	1.23 (1.16 to 1.31)	1.13 (1.07 to 1.19)		NA	NA	NA	NA
2	668	389 (58.2)	1.37 (1.28 to 1.47)	1.18 (1.10 to 1.26)		NA	NA	NA	NA
3 or more	887	602 (67.9)	1.46 (1.38 to 1.54)	1.12 (1.06 to 1.19)		NA	NA	NA	NA
**Loose stools, no.**									
< 5	4 187	1 458 (34.8)	1.00 (ref)	1.00 (ref)		NA	NA	NA	NA
5–7	4 525	2 295 (50.7)	1.41 (1.34 to 1.48)	1.29 (1.23 to 1.36)		NA	NA	NA	NA
8+	1 449	896 (61.8)	1.84 (1.74 to 1.95)	1.54 (1.45 to 1.60)		NA	NA	NA	NA
**Respiratory illness-specific characteristics**									
Indrawing									
No	NA	NA	NA	NA		17 720	7 086 (40.0)	1.00 (ref)	1.00 (ref)
Yes	NA	NA	NA	NA		2 965	1 906 (64.3)	1.52 (1.43 to 1.60)	1.04 (0.98 to 1.10)
Shortness of breath									
No	NA	NA	NA	NA		16 990	6 733 (39.6)	1.00 (ref)	1.00 (ref)
Yes	NA	NA	NA	NA		3 695	2 259 (61.1)	1.53 (1.48 to 1.59)	1.22 (1.18 to 1.27)
Rapid respiratory rate^f^									
No	NA	NA	NA	NA		16 742	6 608 (39.5)	1.00 (ref)	1.00 (ref)
Yes	NA	NA	NA	NA		3 943	2 384 (60.5)	1.53 (1.47 to 1.59)	1.14 (1.09 to 1.19)

CI: confidence interval; MAL-ED: Etiology, Risk Factors, and Interactions of Enteric Infections and Malnutrition and the Consequences for Child Health and Development Project; NA: not applicable; ref: reference; WAMI: water and sanitation, assets, maternal education and income.

^a^ 1201 children had one or more episodes of diarrhoea; 1672 children had one or more episodes of respiratory illness.

^b^ Risk ratio for antibiotic treatment of illness episode by illness characteristic, adjusted for site.

^c^ Adjusted for study site and other illness characteristics.

^d^ Unadjusted averages of the WAMI index are highly confounded by study site and are therefore misleading.

^e^ Fever missing for three episodes; bloody stools missing for one episode.

^f^ Rapid respiration rate (average of two fieldworker-obtained measurements per day) was defined as: > 60 breaths per minute when child is < 60 days old; > 50 breaths per minute at age 60–364 days; and > 40 breaths per minute at age ≥ 365 days.

Slightly fewer diarrhoea episodes in girls (2178/4897; 44.5%) were treated with antibiotics than those in boys (2471/5264; 46.9%; adjusted RR: 0.95; 95% CI: 0.90 to 1.00; Table 3). Higher socioeconomic status was associated with an increase in treatment (RR: 1.19; 95% CI: 1.10 to 1.29).

**Fig. 3:**
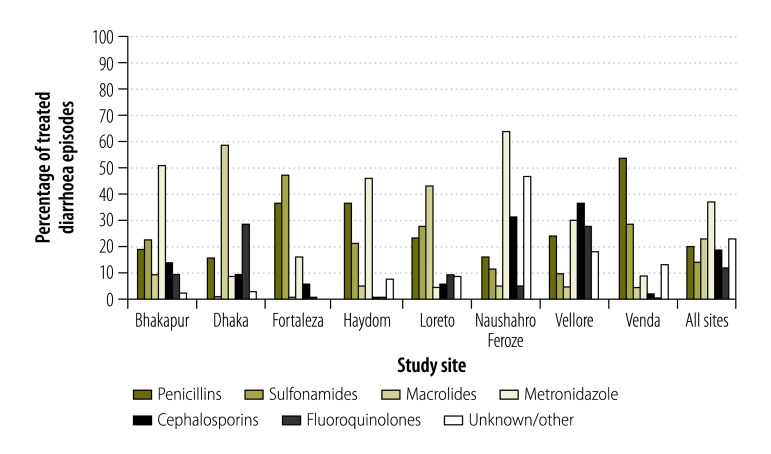
Relative frequency of antibiotic drug classes used in 4649 treated diarrhoea episodes among 1201 children in the MAL-ED birth cohort, 2009–2014

The antibiotic class chosen for diarrhoea treatment varied across and within sites (Fig. 3). Diarrhoea episodes in the sites in Dhaka and Loreto were most often treated with macrolides, while metronidazole was the most common class for diarrhoea treatment in Bhaktapur, Haydom and Naushahro Feroze. Episodes in Fortaleza and Venda were mainly treated with sulfonamides and penicillins. Fluoroquinolones were rarely used for diarrhoea treatment in most sites; their use was most frequent in the South Asian sites of Dhaka and Vellore.

MAL-ED: Etiology, Risk Factors, and Interactions of Enteric Infections and Malnutrition and the Consequences for Child Health and Development Project.

Notes: Distinct antibiotic courses were counted when separated by at least two antibiotic-free days. Courses are included in multiple class categories if more than one drug class was given. Number of treated diarrhoea episodes at study sites: Dhaka (Bangladesh) *n* = 972; Fortaleza (Brazil), *n* = 19; Vellore (India) *n* = 248; Bhaktapur (Nepal) *n* = 326; Naushahro Feroze (Pakistan) *n* = 1899; Loreto (Peru) *n* = 792; Venda (South Africa) *n* =  71; Haydom (United Republic of Tanzania) *n* =  322; all sites *n* = 4649.

Bloody diarrhoea episodes (75/499) were twice as likely to be treated with fluoroquinolones compared with non-bloody episodes (462/9661) (RR adjusted for study site: 2.01; 95% CI: 1.63 to 2.48). Bloody episodes were also 20 to 40% more likely to be treated with macrolides, cephalosporins and metronidazole, and were less likely to be treated with penicillins (51/499 bloody episodes and 870/9661 non-bloody episodes; RR adjusted for study site: 0.57; 95% CI: 0.44 to 0.75).

Diarrhoea episodes among children from higher socioeconomic status were more likely than those occurring among children of lower socioeconomic status to be treated with metronidazole (RR per 0.5 difference in WAMI score: 1.17; 95% CI: 1.06 to 1.30) and macrolides (RR: 1.14; 95% CI: 0.93 to 1.39) and less likely to be treated with penicillins (RR: 0.79; 95% CI: 0.62 to 1.00). The child’s sex did not affect choice of antibiotic class for diarrhoea.

### Respiratory illness treatment

Of 20 685 respiratory illness episodes among 1672 children, 8992 (43.5%) episodes were treated with antibiotics. Use of antibiotics was lowest in Fortaleza (35.5% of 431 episodes) and highest in Haydom (62.8% of 1399 episodes; Table 2).

Fieldworkers confirmed 3943 (19.1%) episodes of respiratory illnesses had signs of acute lower respiratory tract illness. A higher proportion of these episodes (2384; 60.5%) were treated with antibiotics than were episodes of upper respiratory illness (6608/16742; 39.5%; Table 3). Adjusting for site, the risk ratio of antibiotic treatment for acute lower respiratory tract illness compared to upper respiratory illness was 1.53 (95% CI: 1.47 to 1.59).

Respiratory illnesses were significantly more likely to be treated if the episode was of longer duration, and if independently there was fever, shortness of breath or rapid respiratory rate reported (Table 3). Treatment did not vary by age. Similar to diarrhoea treatment, respiratory illness episodes in girls were slightly less likely to be treated with antibiotics (4199/10 019; 41.9%) than those in boys (4793/10 666; 44.9%); after adjusting for study site and episode characteristics, the risk ratio was 0.94 (95% CI: 0.91 to 0.98). Higher socioeconomic status was also associated with a significant but small increase in treatment (adjusted RR: 1.10; 95% CI: 1.03 to 1.17).

**Fig. 4:**
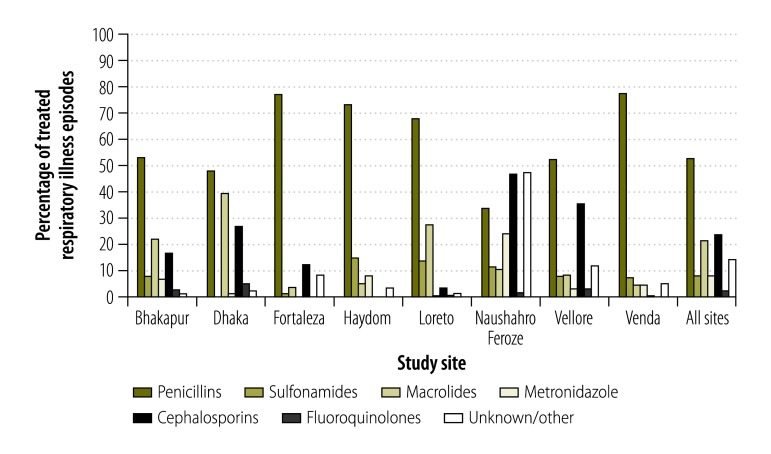
Relative frequency of antibiotic drug classes used in 8992 treated respiratory illness episodes among 1672 children in the MAL-ED birth cohort, 2009–2014

The antibiotic class used for respiratory illness treatment was fairly consistent across sites, with penicillins the most frequently used drug in all sites except Naushahro Feroze (Fig. 4). Cephalosporins were also often chosen in the South Asian sites of Naushahro Feroze, Vellore and Dhaka, while macrolides were also highly used in Dhaka. Because penicillins were almost exclusively used at several sites, a cross-site analysis of antibiotic classes by type of respiratory illness was not possible.

MAL-ED: Etiology, Risk Factors, and Interactions of Enteric Infections and Malnutrition and the Consequences for Child Health and Development Project.

Notes: Distinct antibiotic courses were counted when separated by at least two antibiotic-free days. Courses are included in multiple class categories if more than one drug class was given. Number of treated respiratory illness episodes at study sites: Dhaka (Bangladesh) *n* = 2575; Fortaleza (Brazil), *n* = 153; Vellore (India) *n* = 817; Bhaktapur (Nepal) *n* = 600; Naushahro Feroze (Pakistan) *n* = 2163; Loreto (Peru) *n* = 1490; Venda (South Africa) *n* =  316; Haydom (United Republic of Tanzania) *n* = 878; all sites *n* = 8992.

Among antibiotic-treated respiratory illnesses, higher socioeconomic status was significantly associated with more use of macrolides (RR per 0.5 difference in WAMI score: 1.30; 95% CI: 1.09 to 1.56) and cephalosporins (RR: 1.48; 95% CI: 1.30 to 1.69) and correspondingly less use of penicillins (RR: 0.93; 95% CI: 0.85 to 1.01). There was no association between sex and antibiotic class used for respiratory illness.

### Validation of mothers’ reports

Overall concordance between antibiotics reported in the medical care report forms and caregiver-reported antibiotic use was high 1737 (85.8%) of 2042 forms analysed (Box 1).

Box 1 **Validation of mothers’ reports of antibiotic use in the MAL-ED cohort study, 2009–2014**

Medical care report forms were identified for 13 393 (79.2%) of all 16 913 antibiotic courses, and the validation sample yielded 2024 antibiotic prescriptions from 4409 forms. Concordance between use of antibiotics reported in the medical care report forms and mother-reported antibiotic use was high; on 1737 (85.8%) of forms the antibiotics corresponded with mothers’ reports on the same day. The concordance between antibiotic classes was also high: 95.1% (849/893) for penicillins, 94.8% (329/247) for cephalosporins, 86.6% (265/306) for macrolides, 85.2% (213/250) for metronidazole, 73.4% (177/241) for sulphonamides and 70.0% (42/60) for fluoroquinolones.

## Discussion

Despite substantial heterogeneity, the frequent and early use of antibiotics in these low-resource settings is striking. In the most extreme case, children at the site in Pakistan were exposed to antibiotics on approximately one-fifth of days in their first 6 months of life. Antibiotic usage rates were higher in most sites than that reported for children aged 3 to 24 months in the United States of America in 2010 (0.9 to 1.7 courses per child-year).^42^ Higher antibiotic use in the South Asian sites compared with the African and South American sites is explained by more episodes of diarrhoea and respiratory illnesses as well as a higher proportion of illness episodes treated in this region. Differences in the proportion of episodes treated may be explained by site-specific treatment guidelines and availability of antibiotics. For example, access to antibiotics is less restricted in the South Asian sites,^6^^,^^19^ while drug shortages are common in South Africa.^23^

Illness symptoms were strong drivers of antibiotic treatment for both diarrhoea and respiratory illnesses, demonstrating that treatment decisions were made rationally according to illness severity. However, many episodes of non-bloody diarrhoea (44.2%) and non-acute-lower respiratory-tract illness (39.5%) were treated with antibiotics, contrary to international recommendations against routine use of antibiotics for non-bloody diarrhoea^8^ and upper respiratory tract infections.^43^^,^^44^ These percentages were higher than the overall antibiotic treatment frequency of 37% reported for 17 693 paediatric inpatients from 226 hospitals in 41 countries.^45^ Because only 4.9% of diarrhoea episodes in our study were bloody, almost all antibiotic treatment of diarrhoea (4274/4649 episodes; 91.9%) was for non-bloody episodes, which is inconsistent with treatment guidelines. Similarly, only one-fifth of respiratory infections were fieldworker-confirmed acute lower respiratory tract infection and therefore 73.5% (6608/8992) treated episodes of respiratory illnesses were inconsistent with treatment guidelines.

Conversely, while antibiotic treatment is recommended for dysentery^8^ and acute lower respiratory tract infection,^7^ only 75.2% of diarrhoea episodes with bloody stools and 60.5% of fieldworker-confirmed acute lower respiratory illness episodes were treated with antibiotics. Choice of antibiotic class for diarrhoea treatment was also inconsistent, suggesting diarrhoea treatment guidelines were not clearly followed. Fluoroquinolones and macrolides are recommended by WHO for the treatment of dysentery,^8^ but metronidazole was given most frequently, in more than one-third of dysentery cases. Presence of bloody stools was associated with a higher probability of appropriate treatment with fluoroquinolones and macrolides, but these drugs were still underused.

The lower frequency of treatment among girls compared with boys after adjusting for illness burden and severity indicates that social factors also likely played a role in treatment decisions. Socioeconomic status was associated with frequency of antibiotic treatment as well as the antibiotic classes chosen for both diarrhoea and respiratory illnesses. Increased macrolides and cephalosporins use compared to less penicillins use among families with higher socioeconomic status corresponds to higher prices for these drugs, which may be a barrier to access for low-income families.

This analysis provides a comprehensive description of antibiotic use across eight low-resource country settings, using data reported on every day of the first two years of life, a method which is superior to that of retrospective surveys. Using data on diarrhoea and respiratory illness symptoms, we were able to document treatment frequency and to comment on compliance with international guidelines. Mothers’ reports of antibiotic use ensured that we counted antibiotics taken (not only prescribed) and captured antibiotic use from all sources, including those that would not be included in clinic or prescription records, e.g. those from alternative health-care providers. We found high concordance between mothers’ reports and medical care report forms, as has been previously described,^46^ suggesting mothers’ reporting was reliable.

The study was limited by incomplete details of antibiotic use, including specific drugs given and their formulations, prophylactic versus treatment use, how and where antibiotics were obtained, and the antibiotic class for the courses classified as unknown or other. We also inferred indication for treatment by concurrent illnesses and symptoms reported, without direct reports of the cause of treatment, which limits our ability to determine conclusively the appropriateness of treatment.

Overall, antibiotic use early in life was common, and we found evidence of both overuse for the treatment of non-bloody diarrhoea and upper respiratory tract illnesses, and underuse for the treatment of bloody diarrhoea and acute lower respiratory tract infection. We also found evidence for sex and class differences in access to medicines. Rational antibiotic use programmes and promotion of illness-specific treatment guidelines may have the greatest impact in South Asia, where antibiotic use was highest. Planning of intervention studies involving antibiotic treatment needs to address complex, site-specific variations in use and consider the potentially high baseline frequency of antibiotic use. Further inquiry into the consequences of this highly prevalent exposure among children will be an important contribution to our understanding of child development in low-resource settings.
