# Corrigendum

**DOI:** 10.2471/BLT.17.100217

**Published:** 2017-02-01

In Volume 95, Issue 1, January 2016, page 51 (Fig 1), page 52 (Fig 2) and page 57 (Fig 3 and Fig 4) should have read Bhaktapur: Rogawski ET, Platts-Mills JA, Seidman JC, John S, Mahfuz M, Ulak M, et al. Use of antibiotics in children younger than two years in eight countries: a prospective cohort study. Bull World Health Organ. 2017;95(1):49–61. http://dx.doi.org/10.2471/BLT.16.176123

